# Assessment of the Status of *Cephalanthera longifolia* Populations in Lithuania Derived from a Single-Census Study

**DOI:** 10.3390/plants14132039

**Published:** 2025-07-03

**Authors:** Laurynas Taura, Zigmantas Gudžinskas

**Affiliations:** Laboratory of Flora and Geobotany, State Scientific Research Institute Nature Research Centre, Žaliųjų Ežerų Str. 47, LT-08406 Vilnius, Lithuania

**Keywords:** conservation, endangered species, habitats, plant traits, population demography, species diversity

## Abstract

The study of plant demography is important for identifying ongoing population processes and trends. While single-census studies have limited ability to capture long-term dynamics, they are crucial for establishing baseline data on the status of plant populations. In 2022, four populations of *Cephalanthera longifolia* (Orchidaceae) in Lithuania were studied using a standardised sampling plot method. Within each population, 20 plots were established along a transect. All plant species within each plot were recorded, and their coverage was estimated. Additionally, the height of the plants, the cover of plant debris, and the amount of bare soil in the sampling plot were assessed. Vegetative individuals of *C. longifolia* were dominant across all populations, comprising between 58.7% and 85.1% of all individuals. Combining data from all populations revealed that vegetative individuals accounted for 71.8% of the total population, while generative individuals accounted for the remaining 28.2%. The mean density of individuals in the studied populations ranged from 3.8 ± 2.3 to 11.1 ± 4.3 individuals per square metre. A comparison of plant traits (plant height, inflorescence length, number of flowers in inflorescence, number of fruits set, and number of leaves) was performed between populations. Increased cover of plant debris was found to have the strongest negative effect on the number of individuals. We believe that the demographic type of a population (dynamic, normal or regressive) should be assessed in the context of the life cycle of certain species and their ecological traits, rather than mechanistically. Under reduced light availability, most individuals remained in a vegetative state. Therefore, the ratio of generative to vegetative individuals reflects current habitat conditions rather than long-term population trends.

## 1. Introduction

The study of the demography of plant populations is particularly important for identifying ongoing processes and population trends [1,2]. Such studies can reveal both positive and negative changes in the composition and structure of populations caused by changes in environmental conditions, catastrophic events, human activities, disease, and other factors that threaten populations of endemic, highly specialised or very rare species. These populations may suddenly become vulnerable or even extinct [3,4]. The populations of many widespread plant species are in a state of constant, albeit relatively slow, change, depending on the region, part of the species range or type of habitat [5,6,7,8]. The most reliable information on processes occurring in plant populations can be obtained from long-term studies at permanent research sites. The results of long-term studies have the capability to provide data on changes in habitat, the effects of changing environmental conditions, fluctuations in population, the development of individuals, and reproduction [9,10,11,12]. Single-census population studies, despite their reduced accuracy and inability to detect all processes within populations, offer a comparatively economical option and provide a reasonably objective picture of the status of populations at a given time [13].

The demographic structure of a plant population, which includes data on the density of individuals, the ratio of individuals according to their absolute or relative age (or maturity group), provides comprehensive information on the current state of the population and trends in its development [13,14,15,16]. The results of studies focusing on the demographic structure of populations provide a valuable understanding of the development of invasive species populations, which is necessary for their control, management, and eradication [14,15,17,18,19,20]. Nevertheless, they are also particularly useful for determining the status of populations of rare and endangered plant species, and they form the basis for decisions concerning habitat management and conservation [13,21,22,23].

The number of maturity groups recognised in a study is determined by the biological and ecological characteristics of the target plant species and the objectives of the study [13,15,18,19,20,21,24,25,26]. Individuals are usually divided into several maturity groups, such as seedlings, juveniles, immature, virgin, generative, and senile individuals [19,27]. These groups can be further subdivided into more narrowly defined subgroups such as young generative, mature generative, and old generative individuals [27]. Although classifying individuals into narrowly defined groups is more informative and gives a more accurate indication of the status and trends of the population, it is sometimes impossible to identify the maturity group of an individual using non-destructive methods. Identifying the maturity group of clonal plants is a particularly challenging task [23,28]. This is because it is difficult to define even the boundaries of the individual (genet), with the shoot (ramet) having to be used as the unit of measurement. When studying such plants, they are usually divided into two groups, such as vegetative and generative, non-flowering and flowering, or sterile and fertile individuals [18,23,24,26,29].

Based on the relative proportions of individuals of different life stages, a classification of populations into dynamic, normal, and regressive populations has been proposed [21]. Dynamic populations are characterised by a relatively high proportion of young plants (seedlings, juveniles, and immature individuals). Normal populations have a relatively high proportion of adult (flowering) individuals but still retain a significant number of young individuals. Regressive populations are characterised by a clear dominance of adult stage individuals, particularly large flowering plants, with little evidence of rejuvenation or recruitment. Assessment of demographic composition therefore provides a relatively simple, accessible and efficient tool for identifying ongoing processes in a population when long-term studies are not possible or feasible and a rapid assessment of population status is required [18,21,22,29]. In studies of rare and endangered species, counting the number of reproductive individuals is often used [11,30,31,32], but this count only provides information on the current state of the population and does not allow conclusions to be drawn about long-term population trends or the need for specific conservation measures [32,33].

An accurate determination of the status of a population based on its demographic composition is possible when the development of a given species has been well studied over the entire life cycle, from seedling to mature and senile individual [34,35,36]. Even with a good knowledge of the development of individuals, it is very difficult to accurately assess their maturity group in the wild without damaging the plants. Sometimes the biological characteristics of the species increase the level of uncertainty in identifying the maturity group [37,38]. Mature individuals of terrestrial orchids often do not produce reproductive organs in the year following flowering and fruiting and are almost indistinguishable from immature plants, or they enter a state of secondary dormancy and do not produce above-ground organs [39,40,41].

The population structure of a species is closely related to environmental conditions and habitat characteristics, which in turn are strongly influenced by habitat use and management practices [21,22,24,29]. Studies of *Salvia pratensis* [21] and *Pulsatilla patens* [22] populations have revealed a relationship between ground cover heterogeneity and the proportion of young individuals. Management regimes have also been shown to significantly affect the overall demographic composition of *Salvia pratensis* and *Primula veris* populations [21,24,29]. This suggests that the mosaic of microhabitats plays a crucial role in the plant recruitment [16,21].

The life cycle of terrestrial orchids is intricately linked to their habitats and their relationships with other organisms, such as mycorrhizal fungi and flower pollinators [42,43,44]. During their prolonged juvenile stage, terrestrial orchids develop underground tubers or rhizomes, allowing them to survive in unfavourable conditions such as drought or cold, or even enter a state of secondary dormancy for several years [40,45,46,47]. The pollination of flowers of terrestrial Orchidaceae species frequently depends on specialised insects, which has a significant impact on reproductive success. The dynamics of orchid populations are heavily influenced by environmental stability, natural habitat disturbance, and human activities [47,48]. The long lifespans of many species and their low reproductive rates contribute to population persistence but limit their ability to recover rapidly after a decline [32,38,49]. Habitat destruction and climate change pose significant threats to orchid populations worldwide [41,50].

Although the development and life cycle of most terrestrial orchids is well known, detailed studies of the demographic composition and dynamics of populations are relatively scarce [13,18,26,27,51,52,53,54]. Comprehensive demographic studies of the genus *Cephalanthera* are largely absent across its range, particularly in the northern regions.

*Cephalanthera longifolia* has a wide range and is abundant in some regions of Europe, but in the northern part of the range it is rare and legally protected [55,56,57,58]. The primary factors contributing to this decline are habitat loss and slow reproduction rates [19,21]. However, information on the demographic structure and density of individuals in populations of *C. longifolia*, as well as other *Cephalanthera* species, is scarce and highly fragmented [18,27,43,47,48,58]. The primary challenge in understanding the environmental and anthropogenic factors that contribute to population decline is the absence of consistent data on ongoing processes within populations. This information is essential for selecting effective conservation measures for *Cephalanthera* species [33,58,59]. Therefore, a study was initiated into the structure of populations, density of individuals, morphological traits and relationship of these features to habitat conditions of *C. longifolia* in Lithuania.

The objective of the present single-census study was to determine the structure of *C. longifolia* populations and their dependence on habitat conditions, aiming to select the most appropriate conservation measures. The following research questions were formulated: (a) What was the density of individuals, and how were they distributed in maturity groups in different habitats? (b) What were the effects of habitat conditions on the morphological and reproductive traits of individuals of different maturity groups? (c) What was the relationship between the density of *C. longifolia* individuals and the diversity and abundance of other plant species in the habitat?

## 2. Materials and Methods

### 2.1. Study Species

*Cephalanthera longifolia* is a long-lived myxotrophic perennial with a thick horizontal rhizome. The stem is erect, usually 15–50 cm, occasionally up to 70 cm or even more, with several leafless sheaths in the lower part. The leaves are lanceolate or broadly lanceolate with a pointed tip. The inflorescence is a lax spike with 4–15, occasionally 1–3 and up to 20 or more, flowers. Each flower has a single leaf-like bract, which becomes smaller towards the top of the inflorescence. Flowers are white with a yellowish lip. The flowers are pollinated by insects, mainly by burrowing bees, but do not produce nectar and offer no reward to pollinators. *Cephalanthera longifolia* has a mixed mating system, with successful fruit set after cross-pollination and after self-pollination [58,60,61,62].

The range of C. longifolia includes Europe, western North Africa, and most of Asia, although it is rare or very rare in some regions. It occurs mainly in deciduous and mixed forests, but also in grasslands and rocky mountain slopes. Some authors consider the species to be calciphile, but it can also grow in neutral or slightly acidic soils [63,64]. The species is rare and protected in Lithuania, occurring mainly in the southern and south-eastern parts of the country. The main habitats of C. longifolia are deciduous and mixed forests of various ages, including early successional stages, and grasslands along forest edges [33].

### 2.2. Study Sites

**Figure 1:**
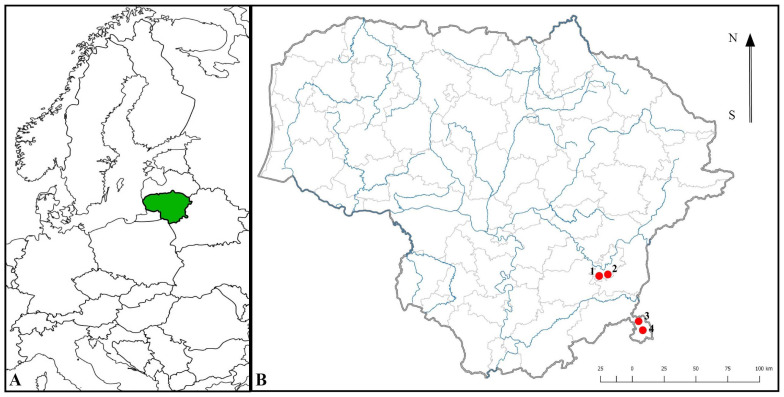
Location of Lithuania in Europe (**A**) and study sites of *Cephalanthera longifolia* populations (**B**): 1. Paneriai; 2. Raisteliai; 3. Stakų Ūta; 4. Katkuškis.

The main requirements for the study site of *C. longifolia* selected for this research were the size of the coenopopulation (minimum area of 0.1 ha), habitat homogeneity (one habitat type) and uniformity of distribution of individuals. Four coenopopulations (hereafter referred to as populations) meeting these criteria were selected in Vilnius city (Paneriai and Raisteliai) and two in Šalčininkai district, Dieveniškės Historical Regional Park, Stakai Landscape Reserve (Katkuškės and Stakų Ūta) (Figure 1). Other populations did not meet the criteria because they were fragmented (individuals formed isolated groups or solitary individuals were widely dispersed), individuals occupied several different habitats or habitats with the target species were highly fragmented.

**Raisteliai**. This study site was selected in a young stand established on an arable land abandoned about 30 years ago [33]. The dominant species in the tree layer was *Betula pendula* and the subdominant species was *Populus tremula* (Table 1). The shrub layer consisted of 18 species and the most abundant species were *Salix caprea*, *Frangula alnus*, *Corylus avellana* and *Sorbus aucuparia*. The herb layer was rather sparse and consisted of a mixture of species characteristic of grasslands and woodlands (*Agrostis capillaris*, *Hepatica nobilis*, *Knautia arvensis*, *Luzula pilosa*, *Maianthemum bifolium*, *Melampyrum nemorosum*, *Veronica chamaedrys*, etc.). The bryophyte layer was sparse and consisted of small patches of *Pleurozium schreberi* and *Dicranum polysetum*.

**Paneriai**. This study site was selected in a premature stand, with dominant trees about 80 years old. The dominant species in the first tree layer was *Betula pendula* and the subdominant species were *Pinus sylvestris* and *Populus tremula*. The second layer of trees was dominated by *Picea abies*. The shrub layer was rather sparse, and the most abundant species were *Quercus robur*, *Frangula alnus*, *Corylus avellana* and *Picea abies*. The herb layer was rather dense and consisted of species characteristic of coniferous forests (*Vaccinium myrtillus*, *Vaccinium vitis-idaea*, *Maianthemum bifolium*, *Luzula pilosa*, *Melampyrum pratense*, etc.) with a significant proportion of species characteristic of deciduous and mixed forests (*Fragaria vesca*, *Hepatica nobilis*, *Stellaria holostea*, *Melampyrum nemorosum*, etc.). The bryophyte layer was quite dense and consisted mainly of *Pleurozium schreberi* and *Dicranum polysetum*.

**Katkuškės**. This study site was chosen in a stand with dominant trees about 60 years old. The dominant species in the first layer of trees were *Picea abies* and *Betula pendula*, and the subdominant species was *Pinus sylvestris*. The second tree layer was dominated by *Picea abies*. The shrub layer was moderately sparse, and the most abundant species were *Picea abies* and *Corylus avellana*. The herb layer was rather sparse and consisted of species characteristic of coniferous forests (*Vaccinium myrtillus*, *Maianthemum bifolium*, *Luzula pilosa*, etc.), while only single individuals of species characteristic of deciduous and mixed forests were recorded. The bryophyte layer was moderately dense and consisted mainly of *Eurhynchium angustirete* and *Hylocomium splendens*.

**Stakų Ūta**. This study site was selected in an early successional stand with dominant trees about 70 years old. The dominant species in the first layer were *Picea abies* and *Betula pendula*, and the subdominant species was *Pinus sylvestris*. The second layer of trees consisted mainly of *Picea abies*. The shrub layer was sparse, and the most abundant species was *Corylus avellana*. The herb layer was dense and consisted of species characteristic of coniferous forests (*Oxalis acetosella*, *Carex digitata*, *Rubus saxatilis*, *Maianthemum bifolium*, *Luzula pilosa*, etc.). The bryophyte layer was rather sparse and consisted mainly of *Pleurozium schreberi* and *Hylocomium splendens*. In the forest plot where the study site was selected, individual trees were felled in 2019 (3 years before the study).

The mean long-term precipitation in the study areas ranged from 685 mm in Vilnius to 701 mm in Šalčininkai, close to the national climate standard of 695 mm [65]. The mean long-term annual temperature in the study areas was 7.2 °C, close to the national climate standard of 7.4 °C. The long-term mean winter temperature ranged from −2.9 °C in Vilnius to −2.6 °C in Šalčininkai, while the mean summer temperature was 17.5 °C in Vilnius and 17.4 °C in Šalčininkai. The long-term mean sunshine duration was 1791 h in both study areas [65].

The soil at all study sites was sandy loam. At Katkuškės and Stakų Ūta, the soil was highly acidic, while at Raisteliai and Paneriai, it was slightly acidic (Appendix A, Table A1). The phosphorus content of the soil varied greatly between the sites, ranging from low at Katkuškės and Stakų Ūta to moderate at Paneriai and high at Raisteliai. The potassium content of the soil was low at Paneriai and Katkuškės and moderate at Raisteliai and Stakų Ūta. Soil total nitrogen content was low at Raisteliai and Katkuškės and moderate at Paneriai and Stakų Ūta. Soil humus content was moderate at all study sites (Appendix A, Table A1).

### 2.3. Field Surveys

Field surveys were conducted at selected sites in the second half of June 2022. In the first step of the assessment, the phytosociological relevés of the plant communities of the study site with *C. longifolia* were made according to the approach of Braun-Blanquet [66], and the cover of plant debris and bare soil was also assessed. The study of the *C. longifolia* population was carried out using the sampling plot method. In each population, 20 sampling plots of 1 m^2^ were arranged in a transect, oriented from south to north. The beginning of the study transect was at least 3 m from the edge of the *Cephalanthera* population to avoid the edge effect and to achieve uniformity of habitat conditions. Sampling plots during the study were delimited by a wooden frame with all sides of 1 m (the frame had measuring scales on each side). Sampling plots were arranged in the transect with the smallest possible spacing (3 cm; the width of the wooden frame).

The tallest plant in the sampling plot was measured, and the mean height of the herb layer at two opposite corners of the plot was measured with a tape measure. All plant species (seedlings and saplings of woody plants, herbaceous plants, and bryophytes) were recorded in each sampling plot, and the area occupied by each species was estimated in square centimetres (1 cm^2^ corresponds to 0.1% of the total sampling plot area). The area of plant debris (dead leaves, branches, bark, etc.) and bare soil (not covered by debris and plants) was also determined in each sampling plot.

All *C. longifolia* individuals recorded in the sampling plots were divided into two groups based on morphological characteristics: vegetative and generative individuals. The group of vegetative individuals included plants with no inflorescence or evidence of its presence in the study year, whereas individuals with an inflorescence containing at least one developed flower (or fruit) were considered as generative individuals.

Annual increment of the *C. longifolia* rhizome has been found to be low, and only shoots growing very close together (less than 5 cm apart) can belong to the same individual (genet). Usually, a single shoot grows from the rhizome, but sometimes the same rhizome can produce a cluster of two or more shoots [60,61]. It is therefore only possible to determine the limits of an individual by digging up the plant or unearthing its rhizome. As we have used only non-invasive and non-destructive methods of study for this protected species, we have considered the shoot (ramet) as the unit of record and refer to it as an individual.

Two traits of each individual (ramet) recorded in the sampling plots were assessed: its height and the number of leaves. The height of an individual was measured with a tape measure to the nearest centimetre from the soil surface to the top. The number of leaves per individual was counted, excluding the lower sheaths, which sometimes had an underdeveloped leaf blade (about 1 cm long), and the bracts, which were sometimes similar in size to the upper leaves of the stem. For generative individuals, three additional traits were assessed: inflorescence length, number of flowers per inflorescence and number of fruits developed. Inflorescence length was measured in centimetres from the lowest axil of the bract to the top of the inflorescence. The number of flowers consisted of all the flowers in the inflorescence, including those that had flowered, those that were still flowering and those that had not yet opened.

### 2.4. Statistical Analysis

The data sets were evaluated using the Shapiro–Wilk test, which revealed that some were not normally distributed (number of individuals in sampling plots, inflorescence length, number of flowers, leaves and developed fruits). Therefore, the data were analysed using non-parametric methods. The results of the descriptive statistics are presented as the mean and standard deviation (mean ± SD). Differences between data sets (populations) were determined using the Kruskal–Wallis H test and differences between pairs of samples were determined using the Dunn post hoc test. Descriptive statistical analyses and comparisons between populations were performed using PAST 5.1 software [67].

Species similarity in the studied populations was assessed using the Jaccard (J) similarity index. This index was calculated using plant species recorded in all sampling plots within each population. To assess the effect of species richness on *C. longifolia* abundance, generalised linear models (GLMs) were applied, using the total number of *C. longifolia* individuals per plot as the response variable. The predictor variables included vascular plant and bryophyte species richness. The models assumed a Gaussian distribution with an identity link. The significance of the models was evaluated using t-statistics and *p*-values. The following habitat and community data were used for the principal component analysis aimed at identifying factors influencing the number of *C. longifolia* individuals: mean plant height, height of the tallest plant, area covered by plant debris, area of bare soil, number of herbaceous plant species and number of moss species in the study plot. For principal component analysis, all variables were centred and scaled, and the correlation matrix was used for analysis. Principal component analysis was performed using R software (version 4.3.2), with data visualisation and testing performed using the ggplot2, vegan and lme4 packages [68].

## 3. Results

### 3.1. Developmental Stages of Individuals

In the four *C. longifolia* populations studied, a total of 577 individuals were recorded and examined in all 80 sampling plots. Of the examined individuals, 414 (71.8%) were vegetative and 163 (28.2%) were generative (Table 2). Although vegetative individuals dominated in all populations, there were clear differences between populations in the ratio of vegetative to generative individuals. The highest proportion (85.1%) of vegetative individuals was found in the Katkuškės population, which was sampled in a dense *Picea abies*-dominated stand (Table 1, Table 2). A similar proportion of vegetative individuals (82.5%) was recorded in the Paneriai population, which was sampled in a relatively dense stand with a lush shrub cover. In the Raisteliai population, which developed in a young *Betula pendula* stand, the proportion of vegetative individuals was significantly lower (61.1%), while the lowest proportion of vegetative individuals (Table 2) was found in the Stakų Ūta population (58.7%), which occupied a sparse *Picea abies* stand with a thin shrub cover.

### 3.2. Density of Individuals

The density of all individuals was significantly different among the four populations (H = 33.78; *p* < 0.001). The highest density of individuals was found in the Raisteliai population (11.1 ± 4.3 individuals/m^2^) and the lowest in the Stakų Ūta population (3.8 ± 2.3 individuals/m^2^). Pairwise comparisons of populations showed that there were no differences in total density of individuals between the Paneriai and Raisteliai (z = 1.83; *p* = 0.068) and Paneriai and Katkuškės (z = 1.55; *p* = 0.122) populations. Stakų Ūta differed significantly (*p* < 0.05) from all populations in the density of individuals.

The density of vegetative individuals of *C. longifolia* was significantly different among the four studied populations (H = 22.69; *p* < 0.001). The highest density of vegetative individuals was found in the Raisteliai population (6.8 ± 3.9 individuals/m^2^), while the lowest density was found in the Stakų Ūta population (2.2 ± 1.8 individuals/m^2^). Pairwise comparisons between populations showed that there were no differences in the density of vegetative individuals between Paneriai and Raisteliai (z = 0.09; *p* = 0.932), Raisteliai and Katkuškės (z = 0.92; *p* = 0.359), and Paneriai and Katkuškės (z = 1.00; *p* = 0.316), while Stakų Ūta was significantly different from all other populations (*p* < 0.01) in the density of vegetative individuals (Table 2).

The studied populations differed more in the density of generative individuals of *Cephalanthera longifolia* than in the density of vegetative individuals (Table 2). Significant differences were found between the densities of generative individuals in the four populations studied (H = 29.42; *p* < 0.001). The highest density of generative individuals was found in the Raisteliai population (4.3 ± 2.4 individuals/m^2^) and the lowest in the Katkuškės population (0.9 ± 1.9 individuals/m^2^). Pairwise comparisons of the populations showed that the Raisteliai population had a significantly (*p* < 0.001) higher density of generative individuals than the other populations, whereas there were no differences between the Paneriai and Katkuškės (z = 1.77; *p* = 0.076) and Paneriai and Stakų Ūta (z = 0.28; *p* = 0.778) populations.

### 3.3. Plant Traits in Studied Populations

**Figure 2:**
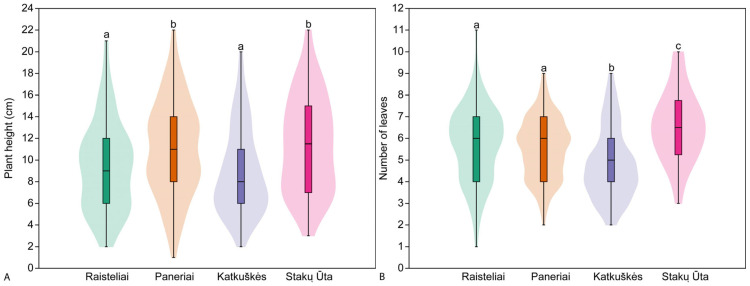
Mean plant height (**A**) and mean number of leaves (**B**) of vegetative *Cephalanthera longifolia* individuals in the studied populations. Different superscript letters indicate significant differences between populations according to the results of pairwise comparison using Dunn’s post hoc test.

The height of vegetative individuals in the studied populations ranged from 1 cm to 22 cm. Significant differences (H = 24.38, *p* < 0.001) in the height of individuals in this group were found between the studied populations. The tallest vegetative individuals were found in the Stakų Ūta population (11.4 ± 4.4 cm), and the shortest in the Katkuškės population (8.8 ± 3.9 cm). Pairwise comparisons showed no significant differences in plant height between the Raisteliai and Katkuškė (z = 1.02, *p* = 0.308) and Paneriai and Stakų Ūta (z = 0.28, *p* = 0.782) populations (Figure 2). The vegetative individuals in the studied populations had between 1 and 11 leaves, and the mean number of leaves per individual ranged from 4.8 ± 1.6 leaves in the Katkuškės population to 6.6 ± 1.6 leaves in the Stakų Ūta population (Figure 2). Significant differences in leaf number were found between all populations studied (H = 38.25, *p* < 0.001). Pairwise comparison of populations showed no significant differences in leaf number of vegetative individuals only between Raisteliai and Paneriai populations (z = 0.91; *p* = 0.364).

**Figure 3:**
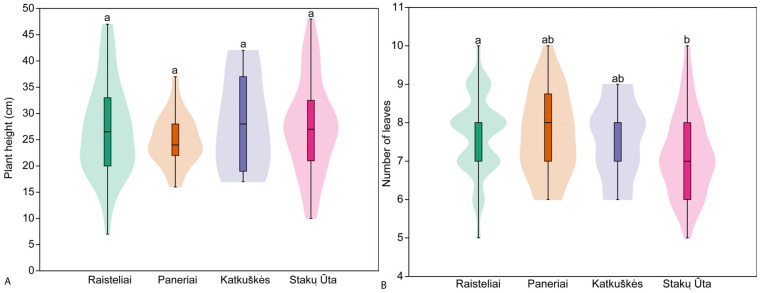
Mean plant height (**A**) and mean number of leaves (**B**) of generative *Cephalanthera longifolia* individuals in the studied populations. Different superscript letters indicate significant differences between populations according to the results of pairwise comparison using Dunn’s post hoc test.

The height of generative individuals in the populations studied ranged from 7 cm to 48 cm. No significant differences (H = 1.48, *p* = 0.685) in the height of generative individuals were found between the populations. The tallest vegetative individuals were found in the Katkuškės population (28.1 ± 9.0 cm) and the shortest in the Paneriai population (24.9 ± 4.7 cm). Pairwise comparisons showed no significant differences in the height of generative individuals between all populations studied (Figure 3). The generative individuals in the studied populations had between 5 and 10 leaves, and the mean number of leaves per individual ranged from 7.2 ± 1.1 leaves in the Stakų Ūta population to 7.7 ± 1.0 leaves in the Raisteliai population (Figure 3). No significant differences (H = 7.16, *p* = 0.051) were found in the number of leaves of generative individuals between the populations. Pairwise comparison of populations showed no significant differences in the number of leaves of generative individuals between all populations (Figure 3), except for a significant difference between Raisteliai and Stakų Ūta populations (z = 2.73; *p* = 0.006).

**Figure 4:**
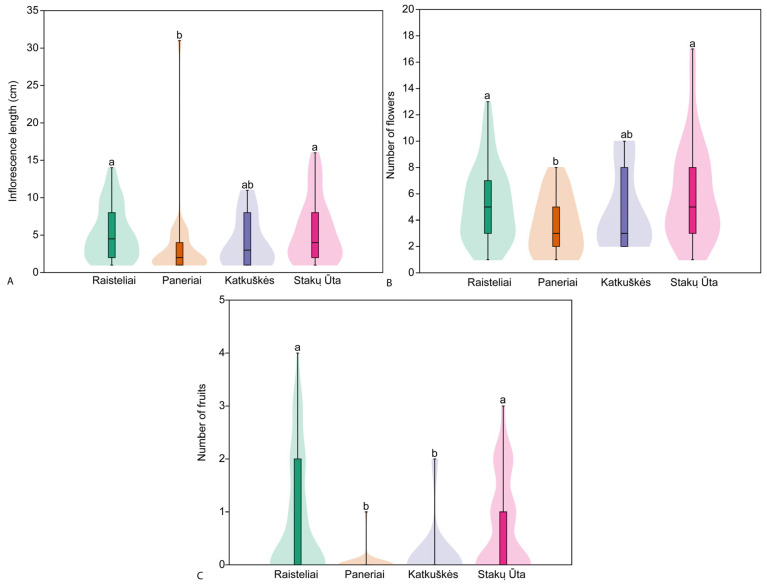
Mean length of inflorescence (**A**), mean number of flowers (**B**) and fruits produced (**C**) of generative *Cephalanthera longifolia* individuals in the studied populations. Different superscript letters indicate significant differences between populations according to the results of pairwise comparison using Dunn’s post hoc test.

The length of the inflorescence in the studied populations ranged from 1 cm to 31 cm, and the mean length of the inflorescence ranged from 3.5 ± 5.7 cm in the Paneriai population to 5.5 ± 4.2 cm in the Stakų Ūta population (Figure 4). Significant differences (H = 13.37, *p* = 0.003) in the length of the inflorescence were found between the studied populations. Pairwise comparison of populations showed significant differences in inflorescence length between Raisteliai and Paneriai populations (z = 3.46; *p* < 0.001) and between Paneriai and Stakų Ūta populations (z = 3.03; *p* = 0.002).

The number of flowers in the inflorescence ranged from 1 to 17 in the studied populations, and the mean number of flowers in the inflorescence ranged from 3.6 ± 2.1 in the Paneriai population to 5.9 ± 3.7 in the Stakų Ūta population (Figure 4). Significant differences (H = 8.33, *p* = 0.037) in the number of flowers in the inflorescence were found between the studied populations. Pairwise comparison of populations showed significant differences in the number of flowers in the inflorescence between Raisteliai and Paneriai populations (z = 2.37; *p* = 0.018) and between Paneriai and Stakų Ūta populations (z = 2.77; *p* = 0.006).

The number of fruits produced by a plant in the studied populations ranged from 0 to 4, and the mean number of fruits produced ranged from 0.0 ± 0.19 in the Paneriai population to 0.8 ± 1.1 in the Stakų Ūta population (Figure 4). Significant differences (H = 13.08, *p* < 0.001) in the number of fruits produced by an individual were found between the populations studied. Pairwise comparison of populations showed significant differences in the number of fruits produced between all populations except Raisteliai and Stakų Ūta (z = 0.07; *p* = 0.942) and between Paneriai and Katkuškės populations (z = 0.40; *p* = 0.687).

### 3.4. Species Richness in Habitats

The highest vascular plant species richness, including *C. longifolia*, in all sample plots studied was found at the Paneriai site (46 species). The species diversity of the Raisteliai site was slightly lower (42 species) than that of the Paneriai site. The Katkuškės site had significantly fewer vascular plant species (32 species), and the Stakų Ūta site had the lowest number of species (29 species). The diversity of bryophytes was the same in all study sites (four species), except for the Katkuškės site (three species).

The highest mean number of species in the sampling plot was found at the Paneriai site (18.4 ± 2.7; range 14–23 species in the sampling plot), while the lowest mean number of vascular plant species was found at the Katkuškės site (8.3 ± 3.0; range 3–14 species). The diversity of vascular plant species was significantly different between the study sites (H = 51.3, *p* < 0.001). Pairwise comparison showed significant differences between all site pairs (*p* < 0.001), except between Raisteliai and Stakų Ūta (U = 132.5, *p* = 0.067).

Comparative analysis of species composition using the Jaccard index (J) indicated that the Paneriai and Stakų Ūta populations were the most similar, sharing 64% of their species (J = 0.64). In contrast, the Katkuškis and Stakų Ūta populations showed the lowest similarity in species composition (J = 0.42). The Paneriai and Raisteliai populations, situated close to each other, shared 57% of species (J = 0.57).

A generalised linear model showed that the total number of *C. longifolia* individuals per plot was not significantly associated with the number of accompanying vascular (t = 1.61, *p* = 0.109) or bryophyte (t = −1.22, *p* = 0.222) species. Similarly, no significant relationships were found between the number of vegetative or generative individuals and vascular plant (t = 1.81, *p* = 0.070 and t = 0.65, *p* = 0.516, respectively) or bryophyte (t = 0.75, *p* = 0.452 and t = −1.85, *p* = 0.065) species richness. These findings suggest that species richness alone does not directly influence the abundance of *C. longifolia* individuals.

### 3.5. Effect of Community Structure

Total vascular plant cover excluding *C. longifolia* in all sampling plots (*n* = 80) was 39.64% (mean 3963.8 ± 2881.5 cm^2^ per plot). Plant debris covered 57.92% of the ground surface area (mean 5791.9 ± 2528.1 cm^2^ per plot), while and bare soil accounted for 2.87% (mean 287.0 ± 1106.6 cm^2^ bare soil per plot).

**Figure 5:**
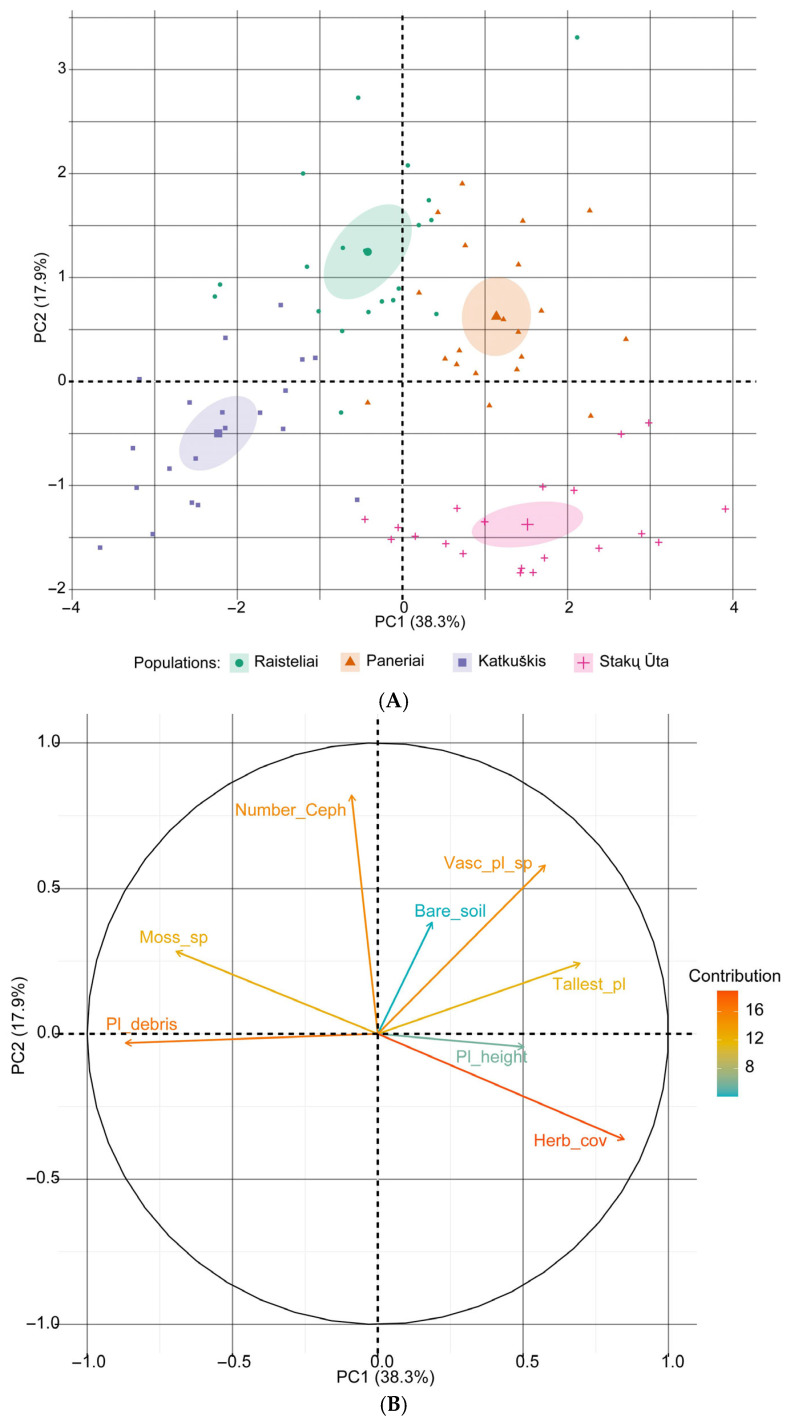
Principal component analysis representing the relationship between the number of *Cephalanthera longifolia* individuals (regardless of maturity stage) and habitat variables: (**A**) scores for the studied populations with 95% confidence limits (ellipses); (**B**) loading plot of the variables contributing to PC1 and PC2. Abbreviations: Number_Ceph: number of *C. longifolia* individuals; Moss_sp: number of moss species; Pl_debris: coverage of plant debris; Herb_cov: coverage of herbs; Pl_height: mean plant height; Tallest_pl: height of the tallest plant; Vasc_pl_sp: number of vascular plant species; Bare_soil: area of bare soil in a sampling plot.

A principal component analysis (Figure 5) based on habitat and community characteristics showed that the first component accounted for 38.3% of the variance (eigenvalue λ = 3.06), while the second component accounted for a further 17.9% (λ = 1.43). Together, the first two components explained 56.2% of the total variance. The strongest negative loadings were associated with the amount of plant debris (−0.868) and the number of bryophyte species (−0.694). This suggests that these variables negatively influence the abundance of *C. longifolia*. Conversely, the area covered by herbaceous plants (excluding *C. longifolia*), the height of the tallest plant and the number of vascular plant species exhibited the strongest positive loadings (0.848, 0.696 and 0.576, respectively), indicating a positive correlation with the abundance of the studied species.

Similar trends were observed when vegetative and generative individuals were analysed separately. For vegetative individuals, the first two components explained 55.2% of the variance (38.5% (λ = 3.07) and 16.7% (λ = 1.34), respectively), whereas for generative individuals, they explained 55.1% (38.3% (λ = 3.07) and 16.8% (λ = 1.34), respectively). The same environmental variables contributed most strongly to variation in both maturity groups, with nearly identical loadings. This consistency suggests that both life stages of *C. longifolia* respond similarly to microhabitat structure, particularly to factors related to the density of the herb layer and plant debris.

## 4. Discussion

### 4.1. Developmental Stages of Individuals

The results of this study showed that vegetative individuals predominated in all populations of *C. longifolia* (ranging from 14.9% to 41.3%). The proportion of vegetative and generative individuals in the pooled data set of all populations studied was 71.8% and 28.2%, respectively. If the studied populations are evaluated in terms of the proportions of immature and mature individuals [21], two of them, Raisteliai and Stakų Ūta, comprising 38.9% and 41.3%, respectively, of generative individuals, should be classified as normal populations. The other two populations, Paneriai and Katkuškės, comprised 17.5% and 14.9% generative individuals, respectively, and should be classified as dynamic populations.

A significantly different distribution of individuals by maturity group was found in 2004–2005 by Ryla and Čiuplys [54] in their study of *C. longifolia* populations in Lithuania. According to the report, in the Šilinė and Klevyčia populations (Šalčininkai district), generative individuals constituted 62% and 54% of all individuals, respectively. In contrast, in the three populations studied in the vicinity of Paneriai, the proportion of generative individuals ranged from 0% to 16%. The authors of the study [54] highlighted that the prevalence of immature individuals is an indication of a healthy population, while a significant proportion of generative individuals in the population signifies its decline and such populations should be classified as regressive.

The results of our study have led us to question whether it is correct to consider a population dominated by generative individuals as a regressive population. Can populations of long-lived clonal plants be assessed using the same criteria as populations of unitary plants [13,21] based on the maturity of the individuals (ramets)? Some authors have already argued several decades ago that the dominance of generative individuals in *C. longifolia* populations indicates optimal habitat conditions for the species rather than its regression [52]. The same phenomenon has been observed in assessment populations of *Epipactis palustris* [53], as well as *Cephalanthera rubra* and other clonal species [18,22].

We believe that the ratio of generative to vegetative individuals in a population cannot be assessed mechanistically, but it is necessary to consider the life cycle and ecological characteristics of the species. Previous studies have shown that in *C. rubra*, under unfavourable conditions or when conditions suddenly deteriorate (a significant increase in shrub abundance and a decrease in light availability), the proportion of generative individuals in the population is either very low [18] or declines significantly over several years [33]. We found that the lowest proportion of generative individuals (14.9% of the total) was in the Katkuškės population, which was in a shaded habitat. Under reduced light availability (coverage of tree and shrub layers was 70% and 30%, respectively), most individuals remained in a vegetative state. Furthermore, during a three-year study in the same population, the natural fruit set rate was significantly lower than in populations with a higher proportion of generative individuals [58]. Therefore, the ratio of vegetative to generative individuals in a population of *C. longifolia*, and probably in populations of other clonal orchids, only indicates the current conditions or status of the population but does not reflect trends in population development. Nevertheless, single-census population assessments are very useful for assessing the status of a population and determining urgent conservation actions. Long-term or periodic assessments of the status of the same populations are needed to assess trends in population development. Long-term or periodic assessment is particularly important when a conservation or management plan is being implemented.

In our opinion, clonal plant populations in which generative individuals (ramets) make up only a small fraction of the total population should be classified as regressive. However, it is unclear at what threshold a population can be considered regressive. Consequently, the Raisteliai and Stakų Ūta populations should be considered normal, whereas the Paneriai and Katkuškės populations should be considered of uncertain status. Further studies involving several plant species are needed to determine the ratio of generative to vegetative individuals required for populations to be classified as normal and dynamic, and the ratio that indicates population regression.

### 4.2. Density of Individuals

There is very little information on the density of *C. longifolia* individuals in populations in Europe or other parts of its range. A study performed on three populations of *C. longifolia* in Ukraine revealed that individual density was generally low, ranging from 0.001 individuals/m^2^ to 0.5 individuals/m^2^ [27]. In Lithuania, the density in the populations studied in 2004–2005 was also low in most cases: in four of the five populations studied, the density of *C. longifolia* ranged from 0.05 individuals/m^2^ to 0.46 individuals/m^2^, and only in one population at the Paneriai site the density was 5.3 individuals/m^2^ [54]. However, in both studies in Lithuania and Ukraine the density was estimated by counting all individuals in the area occupied by the population [27,54]. The results of our study showed that only the results for the Paneriai population can be compared with previous studies. The density of *C. longifolia* in the Paneriai population reported by Ryla and Čiuplys [54] is comparable to the density recorded in this study (8.0 ± 3.5 individuals/m^2^). The highest density of individuals recorded in this study (11.1 ± 4.3 individuals/m^2^) was found in a relatively young population occupying a birch stand that had formed on the site of a former cultivated field [33]. The lowest density of individuals was found in the Stakų Ūta population (3.8 ± 2.3 individuals/m^2^), which occupied a mature forest stand.

Studies using small standardised (m^2^) sampling plots arranged in transects are more accurate because it is possible to count all individuals accurately. In addition, the selected transect can be marked as a monitoring site and the study repeated after a certain time to detect changes. If the entire population area is surveyed, counting individuals takes much more time and some individuals (especially small non-flowering plants) may be missed, especially if the herb cover is dense. The sampling plot method also has some disadvantages. If the population is fragmented, plant density results may be significantly biassed, or the number of sampling plots may need to be large. However, the results obtained using the sampling plot method in a single-census survey are easier to compare because the same number of plots is taken.

### 4.3. Species Richness in Habitats

Although species diversity in plant communities has been extensively studied, little is known about how plant diversity affects the abundance of certain species and, consequently, their population stability [69,70,71]. Among *C. longifolia* populations, total and vascular plant species diversity varied significantly (from 29 to 46 vascular plant species), as did diversity among individual sampling plots (from 3 to 23 vascular plant species). Nevertheless, generalised linear analysis revealed no significant relationships between the total number of individuals or individuals in both studied developmental stages. However, principal component analysis revealed positive relationships between the number of vascular plant species and a negative relationship with the number of bryophyte species and *C. longifolia* individuals in a sampling plot.

The diversity of species in a habitat can be influenced by a variety of factors, including the diversity of ecological conditions, the geographical location of the territory, anthropogenic pressure, and the successional stage of the habitat [72,73]. The highest mean density of individuals, however, was found in the Raisteliai population, which was the youngest of all the studied populations and was located in a formerly cultivated field. The other studied populations were much older [33]. The Paneriai populations had high species diversity and were moderately similar to the Raisteliai population. The high species diversity and status of the *C. longifolia* populations may have been mainly due to light availability in the Paneriai and Raisteliai populations compared to the Katkuškės population (Table 1). Therefore, it can be concluded that population status is determined by an intricate combination of factors, the exact influence of which cannot be determined by a single-census study.

### 4.4. Effect of Community Structure

Principal component analysis revealed consistent patterns in the influence of habitat and community structure on the abundance of *C. longifolia*. The first two principal components explained 56.2% of the total variance in the number of individuals, with the first component accounting for 38.3% alone. The most influential variables were the abundance of plant debris and the number of bryophyte species, which exhibited strong negative loadings. In contrast, herb cover (excluding *C. longifolia*), tallest plant height and vascular plant richness exhibited strong positive loadings. As the principal components analysis showed similar eigenvalues and variable loadings for the total number of individuals and for vegetative and generative individuals separately, it can be assumed that the population status is influenced by structural habitat characteristics rather than random factors.

The abundance of moss cover indicates a low rate of microdisturbance in the habitat. It can be assumed that a dense cover of moss and plant debris acts as a physical barrier, preventing *C. longifolia* seeds from entering the soil and germinating, thereby reducing the reproduction potential of the population [26,49]. It is known that orchid recruitment often depends on fine-scale habitat structure. These findings suggest that the species is most abundant in forest habitats with moderate light availability and reduced plant debris cover, as well as a well-developed herb layer. Therefore, when conducting single-census population studies and assessing their status, more attention should be paid to the condition of the herb layer and ground cover than to species diversity in the habitat.

## 5. Conclusions

The results of a single-census study of four *C. longifolia* populations suggest that the ratio of generative to vegetative individuals reflects the current suitability of the habitat for the species. The lowest proportion of generative individuals (14.9%) was observed in the shaded population. This suggests that reduced light availability leads to a higher proportion of individuals remaining in a vegetative state. This finding calls into question the traditional method of classifying plant populations (dynamic, normal or regressive) based solely on the proportion of maturity groups, particularly for clonal species. Further studies involving several rhizomatous orchid species are needed to determine the threshold at which a population can be considered regressive.

Principal component analysis revealed that certain structural characteristics of the habitat significantly influence the abundance of *C. longifolia* individuals. Increased cover of plant debris and mosses had a strong negative effect on the number of individuals. It is hypothesised that this negative effect is due to dense ground cover creating a physical barrier that hinders seed germination and seedling establishment. In contrast, a well-developed herb layer, as indicated by denser herb cover and the height of the tallest plant, was positively correlated with the abundance of *C. longifolia* individuals.

While species richness in a sampling plot did not exhibit a significant linear relationship with the total number of individuals, the principal component analysis results imply a positive correlation between vascular plant species richness and *C. longifolia* abundance. These findings emphasise the importance of microhabitat structure, such as ground cover and herb layer density, in determining the status of *C. longifolia* populations.

Despite the large number of studies on Orchidaceae species, there is still a lack of knowledge regarding the demographic structure of their populations and how this is affected by environmental conditions. Therefore, studying the demographic structure of endangered or vulnerable species (including Orchidaceae) and how plant traits depend on environmental conditions would greatly benefit the determination of conservation strategies and measures. Large-scale single-census studies should be conducted across a significant portion of the range of each species, with populations selected according to geographical gradient, habitat type or other criteria.

## Figures and Tables

**Table 1:** Cover of vegetation layers and soil surface (%) at the study sites of *Cephalanthera longifolia* populations.

Site	Tree Layers			Shrubs	Herbs	Bryophytes	Plant Debris	Bare Soil
	First	Second	Total					
Raisteliai	30	10	30	50	50	10	40	0
Paneriai	40	20	50	40	70	60	40	1
Katkuškės	40	60	70	30	30	40	60	5
Stakų Ūta	30	30	40	20	80	30	30	3

**Table 2:** The total number of individuals, their number by maturity groups and the density (individuals/m^2^) of *Cephalanthera longifolia* individuals in the studied populations. Different superscript letters indicate significant differences between populations according to the results of pairwise comparison using Dunn’s post hoc test.

Population	Total Number	Mean ± SD	Vegetative	Mean ± SD	Generative	Mean ± SD
Raisteliai	221	11.1 ± 4.3 ^a^	135	6.8 ± 3.9 ^a^	86	4.3 ± 2.4 ^a^
Paneriai	160	8.0 ± 3.5 ^ac^	132	6.6 ± 3.6 ^a^	28	1.4 ± 1.2b ^cd^
Katkuškės	121	6.1 ± 2.6 ^bc^	103	5.2 ± 2.6 ^a^	18	0.9 ± 1.9 ^c^
Stakų Ūta	75	3.8 ± 2.3 ^d^	44	2.2 ± 1.8 ^b^	31	1.5 ± 1.3 ^d^
Pooled	577	7.2 ± 4.2	414	5.2 ± 3.5	163	2.0 ± 2.2
Percentage	100%		71.8%		28.2%	

## Data Availability

All summarised data are presented in the article, and the authors can provide the original research data upon individual request and under agreed conditions.

## Appendix A

**Table A1:** Agrochemical properties of soils at *Cephalanthera longifolia* population study sites.

Site	pH	P_2_O_5_ (mg/kg)	K_2_O (mg/kg)	N _total_ (mg/kg)	Humus (%)
Raisteliai	5.2	172	82	1.02	2.01
Paneriai	5.7	147	58	1.39	1.81
Katkuškės	4.2	13	37	0.80	2.00
Stakų Ūta	4.4	50	78	1.21	2.61

## References

[1] Selwood K.E., McGeoch M.A., Mac Nally R. (2015). The effects of climate change and land-use change on demographic rates and population viability. Biol. Rev..

[2] Iler A.M., CaraDonna P.J., Forrest J.R., Post E. (2021). Demographic consequences of phenological shifts in response to climate change. Annu. Rev. Ecol. Evol. Syst..

[3] Knapp W.M., Frances A., Noss R., Naczi R.F.C., Weakley A., Gann G.D., Baldwin B.G., Miller J., McIntyre P., Mishler B.D. (2021). Vascular plant extinction in the continental United States and Canada. Conserv. Biol..

[4] Mehrabian A.R., Sayadi S., Kuhbenani M.M., Yeganeh V.H., Abdoljabari M. (2020). Priorities for conservation of endemic trees and shrubs of Iran: Important Plant Areas (IPAs) and Alliance for Zero Extinction (AZE) in SW Asia. J. Asia-Pac. Biodivers..

[5] Vegas-Vilarrúbia T., Rull V., Montoya E., Safont E. (2011). Quaternary palaeoecology and nature conservation: A general review with examples from the Neotropics. Quat. Sci. Rev..

[6] Pimm S.L., Jenkins C.N., Abell R., Brooks T.M., Gittleman J.L., Joppa L.N., Raven P.H., Roberts C.M., Sexton J.O. (2014). The biodiversity of species and their rates of extinction, distribution, and protection. Science.

[7] Essl F., Dullinger S., Rabitsch W., Hulme P.E., Pyšek P., Wilson J.R., Richardson D.M. (2015). Historical legacies accumulate to shape future biodiversity in an era of rapid global change. Divers. Distrib..

[8] Eichenberg D., Bowler D.E., Bonn A., Bruelheide H., Grescho V., Harter D., Jandt U., May R., Winter M., Jansen F. (2021). Widespread decline in Central European plant diversity across six decades. Glob. Change Biol..

[9] Franklin J., Serra-Diaz J.M., Syphard A.D., Regan H.M. (2016). Global change and terrestrial plant community dynamics. Proc. Natl. Acad. Sci. USA.

[10] Álvarez-Yépiz J.C., Búrquez A., Martínez-Yrízar A., Dovciak M. (2019). A trait-based approach to the conservation of threatened plant species. Oryx.

[11] Kaya O.F., Ertekin A.S. (2024). Evaluation of the taxonomy and conservation status of *Podonosma sintenisii* (Boraginaceae). Botanica.

[12] Turco A., Wagensommer R.P., Medagli P., D’Emerico S., Ippolito F., Scordella G., Albano A. (2025). *Centaurea pumilio* (Asteraceae): Conservation status, threats and population size of a critically endangered species in Italy. Plants.

[13] Oostermeijer J.G.B., Hartman Y. (2014). Inferring population and metapopulation dynamics of *Liparis loeselii* from single-census and inventory data. Acta Oecol..

[14] Hüls J., Otte A., Eckstein R.L. (2007). Population life-cycle and stand structure in dense and open stands of the introduced tall herb *Heracleum mantegazzianum*. Biol. Invasions.

[15] Pergl J., Hüls J., Perglová I., Eckstein R.L., Pyšek P., Otte A. (2007). Population dynamics of *Heracleum mantegazzianum*. Ecology and Management of Giant Hogweed (Heracleum mantegazzianum).

[16] Crone E.E., Menges E.S., Ellis M.M., Bell T., Bierzychudek P., Ehrlén J., Williams J.L. (2011). How do plant ecologists use matrix population models?. Ecol. Lett..

[17] Meekins J.F., McCarthy B.C. (2002). Effect of population density on the demography of an invasive plant (*Alliaria petiolata*, Brassicaceae) population in a southeastern Ohio forest. Am. Midl. Nat..

[18] Gudžinskas Z., Žalneravičius E., Norkevičienė E., Obelevičius K., Mildažienė V., Stankevičienė K., Balsevičius A., Narijauskas R., Gudžinskas Z., Žalneravičius E., Norkevičienė E., Obelevičius K. (2016). State and dynamics of the protected plant species in the south-western Lithuania under conditions of the climate changes. Conservation of Botanical Diversity in South-Western Lithuania.

[19] Taura L., Gudžinskas Z. (2020). Life stages and demography of invasive shrub *Cytisus scoparius* (Fabaceae) in Lithuania. Botanica.

[20] Gudžinskas Z., Kazlauskas M. (2022). The first record of *Heracleum mantegazzianum* Sommier & Levier (Apiaceae) in Lithuania. BioInvasions Rec..

[21] Hegland S.J., Van Leeuwen M., Oostermeijer J.G.B. (2001). Population structure of *Salvia pratensis* in relation to vegetation and management of Dutch dry floodplain grasslands. J. Appl. Ecol..

[22] Kalliovirta M., Ryttäri T., Heikkinen R.K. (2006). Population structure of a threatened plant, *Pulsatilla patens*, in boreal forests: Modelling relationships to overgrowth and site closure. Biodivers. Conserv..

[23] Rasimavičius M., Naujalis J.R., Gudžinskas Z. (2023). Structure of *Equisetum variegatum* (Equisetaceae) populations in natural and anthropogenic habitats. Botanica.

[24] Brys R., Jacquemyn H., Endels P., Hermy M., De Blust G. (2003). The relationship between reproductive success and demographic structure in remnant populations of *Primula veris*. Acta Oecol..

[25] Colas B., Kirschner F., Riba M., Olivieri I., Mignot A., Imbert E., Fréville H. (2008). Restoration demography: A 10-year demographic comparison between introduced and natural populations of endemic *Centaurea corymbosa* (Asteraceae). J. Appl. Ecol..

[26] Jermakowicz E., Brzosko E. (2016). Demographic responses of boreal-montane orchid *Malaxis monophyllos* (L.) Sw. populations to contrasting environmental conditions. Acta Soc. Bot. Pol..

[27] Pylypiv Y. (2020). Structural features of the Orchidaceae populations. Sci. Horiz..

[28] Cook R.E. (1983). Clonal plant populations: A knowledge of clonal structure can affect the interpretation of data in a broad range of ecological and evolutionary studies. Am. Sci..

[29] Brys R., Jacquemyn H., Endels P., De Blust G., Hermy M. (2004). The effects of grassland management on plant performance and demography in the perennial herb *Primula veris*. J. Appl. Ecol..

[30] Kazlauskas M., Taura L., Gudžinskas Z. (2022). Current state of critically endangered *Neotinea ustulata* (Orchidaceae) in Lithuania and report on a new record of the species. Botanica.

[31] El Karmoudi Y., Libiad M., Fahd S. (2025). Diversity and conservation strategies of wild Orchidaceae species in the West Rif region (northern Morocco). Botanica.

[32] Štípková Z., Kindlmann P. (2025). Distribution of population sizes in metapopulations of threatened organisms—Implications for conservation of orchids. Plants.

[33] Taura L., Gudžinskas Z. (2024). *Cephalanthera longifolia* and *Cephalanthera rubra* (Orchidaceae) in Lithuania: Analysis of distribution, population dynamics and conservation issues. Botanica.

[34] Cabral J.S., Schurr F.M. (2010). Estimating demographic models for the range dynamics of plant species. Glob. Ecol. Biogeogr..

[35] Salguero-Gómez R., Siewert W., Casper B.B., Tielbörger K. (2012). A demographic approach to study effects of climate change in desert plants. Philos. Trans. R. Soc. B.

[36] Ehrlén J., Morris W.F. (2015). Predicting changes in the distribution and abundance of species under environmental change. Ecol. Lett..

[37] Kitajima K., Fenner M., Fenner M. (2000). Ecology of seedling regeneration. Seeds: The Ecology of Regeneration in Plant Communities.

[38] Whigham D.F., Willems J.H., Dixon K.W., Barrett S.P., Cribb P. (2003). Demographic studies and life-history strategies of temperate terrestrial orchids as a basis for conservation. Orchid Conservation.

[39] Gregg K.B. (2011). Recovery from bud disappearance explains prolonged dormancy in *Cleistes bifaria* (Orchidaceae). Am. J. Bot..

[40] Juárez L., Montaña C., Franco M. (2014). The viability of two populations of the terrestrial orchid *Cyclopogon luteoalbus* in a fragmented tropical mountain cloud forest: Dormancy delays extinction. Biol. Conserv..

[41] Shefferson R.P., Mizuta R., Hutchings M.J. (2017). Predicting evolution in response to climate change: The example of sprouting probability in three dormancy-prone orchid species. R. Soc. Open Sci..

[42] Chauhan P., Attri L. (2024). Mycorrhizal associations in orchids: A review. Asian J. Biol. Life Sci..

[43] Mawinei N., Paramitha Q. (2024). Comparative anatomy of roots and leaves in epiphytic and terrestrial orchids: Insights into adaptations and ecological strategies. Law Econ..

[44] Ray H., Gillett-Kaufman J. (2022). By land and by tree: Pollinator taxa diversity of terrestrial and epiphytic orchids. J. Pollinat. Ecol..

[45] Shefferson R.P. (2006). Survival costs of adult dormancy and the confounding influence of size in lady’s slipper orchids, genus *Cypripedium*. Oikos.

[46] Rock-Blake R., McCormick M.K., Brooks H.E.A., Jones C.S., Whigha D.F. (2017). Symbiont abundance can affect host plant population dynamics. Am. J. Bot..

[47] Shefferson R.P., Sandercock B.K., Proper I., Beissinger S.R. (2001). Estimating dormancy and survival of a rare herbaceous perennial using mark-recapture models. Ecology.

[48] Kindlmann P., Willems J.H., Whigham D.F. (2002). Trends and Fluctuations and Underlying Mechanisms in Terrestrial Orchid Populations.

[49] Shefferson R.P., Jacquemyn H., Kull T., Hutchings M.J. (2020). The demography of terrestrial orchids: Life history, population dynamics and conservation. Bot. J. Linn. Soc..

[50] Wraith J., Pickering C. (2019). A continental scale analysis of threats to orchids. Biol. Conserv..

[51] Willems J.H. (1982). Establishment and development of a population of *Orchis simia* (Lamk.) in The Netherlands, 1972 to 1981. New Phytol..

[52] Kolon K., Dudzic J., Krawczyk J., Sadowska A. (1993). Ekologiczna charakterystyka populacji *Cephalanthera longifolia* (L.) Fritsch na Łysej Górze k. Rząśnika. Acta Univ. Wratislav. Pr. Bot..

[53] Kolon K., Krawczyk J., Krawczyk A. (1995). Charakterystyka ekologiczna populacji *Epipactis palustris* (L.) Crantz znad jeziora Pomorze w Puszczy Augustowskiej. Acta Univ. Wratislav. Pr. Bot..

[54] Ryla M., Čiuplys R., Czyżewska K., Hereźniak J. (2005). Populations of *Cephalanthera longifolia* (L.) Fritsch in Lithuania. Biodiversity in Relation to Vegetation Zones in Europe.

[55] Brzosko E., Wróblewska A. (2003). Genetic variation and clonal diversity in island *Cephalanthera rubra* populations from the Biebrza National Park, Poland. Bot. J. Linn. Soc..

[56] Sundberg S. (2017). Present status for *Cephalanthera rubra* and *Chimaphila umbellata* in Sweden. Sven. Bot. Tidskr..

[57] Žalneravičius E., Rašomavičius V. (2021). *Cephalanthera rubra*, (L.) Rich. Red Data Book of Lithuania: Animals, Plants, Fungi.

[58] Taura L., Gudžinskas Z. (2024). What factors determine the natural fruit set of *Cephalanthera longifolia* and *Cephalanthera rubra*?. Diversity.

[59] Pykälä J. (2019). Habitat loss and deterioration explain the disappearance of populations of threatened vascular plants, bryophytes and lichens in a hemiboreal landscape. Glob. Ecol. Conserv..

[60] Püttsepp Ü., Kull T. (1997). *Cephalanthera longifolia* and *Cephalanthera rubra* in Estonia. Bot. Lith..

[61] Abadie J.C., Püttsepp Ü., Gebauer G., Faccio A., Bonfante P., Selosse M.-A. (2006). *Cephalanthera longifolia* (Neottieae, Orchidaceae) is mixotrophic: A comparative study between green and nonphotosynthetic individuals. Can. J. Bot..

[62] Taura L., Gudžinskas Z. (2025). Effect of simulated autogamy and allogamy on the success of *Cephalanthera longifolia* and *Cephalanthera rubra* (Orchidaceae) fruit set. Diversity.

[63] Dafni A. (1979). Orchids of Israel: Notes on distribution, ecology and local variation. Mitt. Arb. Heim. Orchid. Baden-Württ..

[64] Delforge P. (2005). Guide des orchidées d’Europe, d’Afrique du Nord et du Proche-Orient.

[65] Galvonaitė A., Valiukas D., Kilpys J., Kitrienė Z., Misiūnienė M. (2013). Climate Atlas of Lithuania.

[66] Braun-Blanquet J. (1964). Pflanzensoziologie: Grundzüge der Vegetationskunde. Dritte Auflage.

[67] Hammer Ø., Harper D.A.T., Ryan P.D. (2001). PAST: Paleontological Statistics Software Package for Education and Data Analysis. Palaeontol. Electron..

[68] R Core Team (2023). R: A Language and Environment for Statistical Computing.

[69] Thein S., Roscher C., Schulze E.-D. (2008). Effects of trait plasticity on aboveground biomass production depend on species identity in experimental grasslands. Basic. Appl. Ecol..

[70] Barry K.E., de Kroon H., Dietrich P., Harpole S.W., Roeder A., Schmid B., Clark A.T., Mayfield M.M., Wagg C., Roscher C. (2019). Linking species coexistence to ecosystem functioning—A conceptual framework from ecological first principles in grassland ecosystems. Adv. Ecol. Res..

[71] Roeder A., Schweingruber F.H., Ebeling A., Eisenhauer N., Fischer M., Roscher C. (2021). Plant diversity effects on plant longevity and their relationships to population stability in experimental grasslands. J. Ecol..

[72] Pacifici M., Foden W.B., Visconti P., Watson J.E., Butchart S.H., Kovacs K.M., Scheffers B.R., Hole D.G., Martin T.G., Akçakaya H.R. (2015). Assessing species vulnerability to climate change. Nat. Clim. Change.

[73] Feng J.Q., Zhang F.P., Huang J.L., Hu H., Zhang S.B. (2021). Allometry between vegetative and reproductive traits in orchids. Front. Plant Sci..

